# Management of Clozapine Titration in the Setting of Cardiac Comorbidities

**DOI:** 10.7759/cureus.19257

**Published:** 2021-11-04

**Authors:** Theja Bhamidipati, Krishna Divadeenam

**Affiliations:** 1 Department of Psychiatry and Behavioral Sciences, Kansas City Veterans Affairs Medical Center/Kansas City University, Kansas City, USA; 2 Department of Psychiatric and Behavioral Sciences, Kansas City Veterans Affairs Medical Center, Kansas City, USA

**Keywords:** schizophrenia, crp, myocarditis, psychiatry, clozapine

## Abstract

Treatment-resistant schizophrenia is commonly treated by the initiation of Clozapine therapy. Clozapine (Clozaril) has a wide side effect profile with significant mortality stemming from early myocarditis or late cardiomyopathy. This risk profile is complicated in those with preexisting comorbidities.

A 67-year-old male with a decade-long history of paranoid schizophrenia. His previous treatment regimen consisted of a combination of Haloperidol-Decanoate, Aripiprazole, and Olanzapine. On his most recent admission, the patient presented with an acute exacerbation of his schizophrenia with incontinence, agitation, and difficulty following commands. Due to the refractory nature of his symptoms, Clozapine therapy was initiated. During this time serial C-reactive protein (CRP) measurements increased markedly. This increase was seen in the context of worsening lower leg edema and air hunger. Clozapine taper was held, and the medical team was consulted. The consultation resulted in an echocardiogram showing signs of diastolic failure with an unknown etiology. Subsequent CT chest, however, ruled out any pericardial pathology and eliminated suspicion for clozapine-induced myocarditis. Clozapine taper was then restarted.

When beginning clozapine in a patient with underlying cardiac risk factors, it is paramount to take into consideration the patient’s baseline cardiopulmonary function. This report outlines the necessity of a baseline echocardiogram for patients with severe cardiac comorbidities. This in turn may have prevented a four-day delay of clozapine titration. Earlier and more frequent CRP measurements titration would have also guided clinical assessment as well. Furthermore, this case stresses the larger implications of investigating medical comorbidities among patients presenting on a psychiatric unit.

## Introduction

Treatment-resistant schizophrenia is defined as persistence of moderate symptoms with functional impairment that has been refractory to either one or more previous treatments for greater than six weeks [1]. Clozapine is a second-generation atypical antipsychotic that antagonizes both dopamine type 2 (D2) receptors and serotonin type 2A (5HT2A) receptors [2]. Once proper trials of previous treatments have been confirmed for treatment of schizophrenia, initiation with clozapine therapy can be considered pending the evaluation of the patient’s absolute neutrophil count (ANC) [3]. With an ANC greater than 1,500 cells/microliter, a clozapine taper can be started at dosages of 12.5mg/day and titrated up every two days in an elderly patient [3]. During the subsequent titration, the patient should be monitored for the most common causes of early mortality which include adynamic ileus [4] and early myocarditis [5]. The latter adverse effect is poorly understood and thought to be an IgE-mediated reaction [6]. More so, it is particularly difficult to monitor as a definitive diagnosis is only achievable with biopsy which is rarely done antemortem. However, nonspecific inflammatory markers can be combined with clinical suspicion to diagnose early myocarditis. Thus, in the absence of a biopsy, clozapine-induced myocarditis becomes a clinical diagnosis. Unfortunately, the ambiguity surrounding this diagnosis is amplified in patients with preexisting cardiopulmonary complications. Elevation in nonspecific markers such as CRP does not distinguish it from a drug-related pathology or a more chronic disease state. More so, CRP has been shown to become elevated in situations of metabolic stress which is common among elderly patients [7]. Thus, in the setting of a patient with multiple comorbidities the diagnosis of myocarditis becomes difficult and requires establishing a functional baseline for the patient. This case report outlines the management and workup of a patient with preexisting cardiac comorbidities who has begun treatment with clozapine for treatment-resistant schizophrenia.

## Case presentation

The patient is a 67-year-old man with a long-standing history of schizophrenia with catatonic features and previous episodes of hyponatremia. First diagnosed over a decade ago, he has been able to achieve a baseline level of lucidity and mental acuity using a complex treatment regimen that included Aripiprazole 30mg (Abilify) and Quetiapine 150mg (Seroquel). However, due to worsening symptoms and noncompliance, 100mg Haloperidol-Decanoate injection was added every three weeks. Despite this modification, He still showed signs of decompensation as recently as three months prior to his current admission. At this time point, the long-acting injectable was increased in dosage to 150mg every three weeks. This dose adjustment ultimately proved ineffective as he was admitted later for acute agitation, incontinence, and difficulty being redirected, all of which represented significant deviations from his previous baseline.

On admission, he presented with acute decompensation of his schizophrenia. He was alert and orientated only to a person, incontinent of bladder and bowel, unable to be directed, and exhibited signs of bilateral ankle restlessness. The mental status exam showed blunted affect, mumbled speech with an irregular rhythm, low tone, and poverty of speech. Physical exam showed a shuffling gait and mild lower extremity edema. Chart review showed the patient had a history of severe hyponatremia at 122mEq/L (normal 135mEq/L-145mEq/L), which resulted in discontinuation of the Aripiprazole and Quetiapine. At the current admission date, the patient’s sodium level fluctuated between 131 and 134mEq/L which became his new baseline. The most recent Haloperidol-Decanoate injection was two days prior to arrival. However, due to the persistent nature of his disease, Clozapine was initiated after submitting an appropriate ANC count to the Risk Evaluation and Mitigation Strategy. EKG, troponin and CRP were also measured prior to initiation and all were within normal limits. 12.5mg of Clozapine was initiated with plans to titrate up 12.5mg per day until the therapeutic dose of 300mg was reached [7]. Within a few days of beginning clozapine, the patient’s baseline mental acuity markedly increased. Per nursing reports, the patient was more active and had goal-directed conversations. Larger periods of lucidity were noticed, and the patient was beginning to form longer sentence structures and exhibit more complex thoughts including the use of metaphors and formulate arguments.

**Figure 1:**
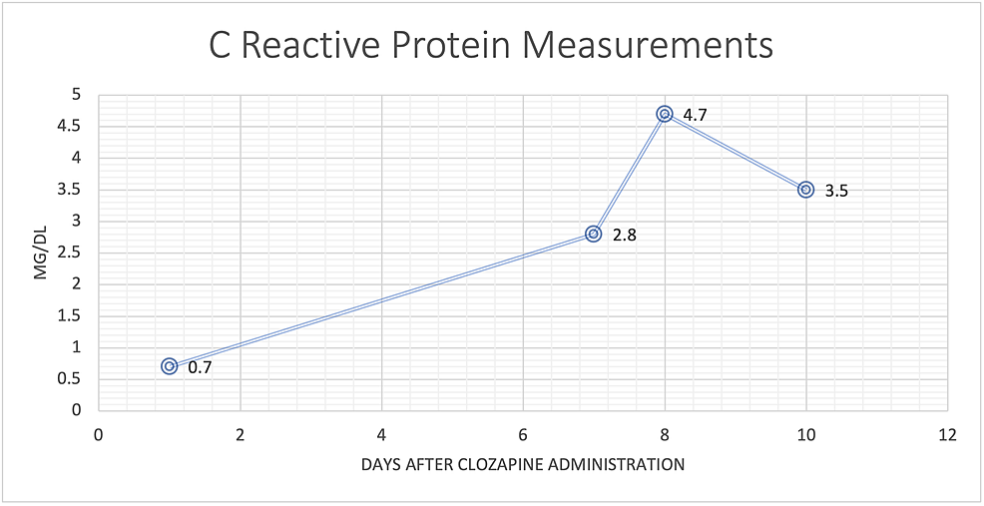
C-reactive protein measurements of the patient

On day 7 after initiation, routine evaluation of CRP (Figure 1) showed an elevation to 2.8mg/dL (normal <1mg/dL), which was markedly increased from 0.7mg/dL on day 1. Simultaneously, the patient developed worsening lower leg edema and shortness of breath. The mental status exam, however, continued to show improvement in the patient’s thought process. This was largely evident by prolonged conversations between the patient and his caregiver.

However, immediate suspicion for clozapine-induced myocarditis was raised and the clozapine taper was held at 50mg (12.5mg in the morning [qAM] and 37.5mg at night [qHS]). Medicine and cardiology consults were placed to determine the etiology of the lower extremity edema in the setting of rising CRP. Chest x-ray showed cardiomegaly and pulmonary infiltrates which did not rule out concern for clozapine-induced myocarditis but did lower the initial suspicion. An echocardiogram was ordered due to the suspicion of heart failure. In the interim, the patient was started on daily furosemide 40mg and a two-liter fluid restriction was placed.

On day 8, CRP was increased to 4.7mg/dL increasing the likelihood of myocarditis. The echocardiogram however showed a normal ejection fraction of 65% (normal 50%-70%) with aspects of diastolic heart failure. This fact, taken in the context of the newly ordered troponin of 0.12ng/ml (normal 0.00ng/mL-0.40ng/mL), offered enough reassurance to restart the clozapine taper. Clozapine was slowly re-titrated up in intervals of 12.5mg every two days. Daily furosemide and fluid restriction were continued. CT chest was ordered to further investigate the etiology of the patient’s symptoms. Discussion with the caregiver established that the lower extremity edema was in fact a longstanding issue. The mental status exam was consistent with previous exams and showed that the patient was at a new improved baseline since beginning clozapine.

On day 10 after clozapine initiation, CRP was decreased down to 3.5mg/dL and CT chest did not show any indication of pericardial pathology or signs of excess intravascular fluid. Furosemide treatment noticeably decreased lower extremity edema and symptoms of shortness of breath were well managed with albuterol/tiotropium inhalers. At this time more aggressive clozapine titration was begun in order to more quickly reach a target dose of 300mg. Titration beginning at 25mg was done every day until a therapeutic dose of 100mg qAM and 200mg qHS is reached approximately 19 days after the therapy was begun.

On day 24 after clozapine initiation, the patient received his next scheduled 150mg Haldol-Decanoate injection.

## Discussion

This report outlines the management and workup of a patient with preexisting cardiac comorbidities who is simultaneously being treated with clozapine. Daily trending lab work with multiple radiographic imaging modalities offered the ability to fine-tune the patient’s treatment to maximize efficiency and safety. However, this approach is difficult to manage in an outpatient setting or in a resource-limited environment. Nonetheless, the considerations taken by this report can have implications in all settings. Currently, only symptomatic management and clozapine discontinuation are recommended for any suspicion of clozapine injury [8]. Continuous cardiac monitoring is used to deliberate whether reintroduction of clozapine is possible [8].

The first highlight of this case is that recommended screening to be done on all patients to receive clozapine. While the clozapine registry only requires weekly submission of ANC for the first six weeks, it is up to the clinician to determine what other diagnostic steps are required to maximize safety for the patient. In the case of this patient, his preexisting diastolic heart failure would warrant not only additional lab work in the forms of troponin, BNP, and CRP but also an increased frequency of these labs more than once every week. For example, serial cardiac labs done every three days would have offered earlier insight into any decompensation and may have resulted in earlier intervention. More importantly, a baseline echocardiogram prior to beginning the medication would have shown the diastolic heart failure and thus contextualized the future abnormal lab work. While elevations in CRP are not historically directly associated with heart failure there is an association between the two [9]. Taken together, these steps would have allowed the patient to reach optimal levels of clozapine at a faster rate which in turn would have decreased his hospital course.

However, there is a broader implication associated with this report. Establishing a baseline requires good history taking and chart review. It was not until collateral information was collected from the caregiver that the lower leg edema was determined to be a preexisting chronic condition. At that time, the worry for an acute cardiac process was substantially ruled out. If for instance, this information was gathered earlier, there would have not been stagnation in clozapine titration and the patient would have received his therapeutic dose much earlier.

On the most recent exam, he showed increased mental acuity and verbal articulation. His episodes of incontinence had been resolved. He is able to be easily directed and follows goal-orientated tasks. He offers much greater insight into his situation. He will be discharged back to his long-term care facility under the guardianship of his caretaker with outpatient follow-up for clozapine monitoring and a plan to taper down his long-acting injectable over the course of six to nine months.

## Conclusions

When beginning clozapine in a patient with underlying cardiac risk factors, it is paramount to take into consideration the patient’s baseline cardiopulmonary function. This report outlines the necessity of a baseline echocardiogram for patients with severe cardiac comorbidities. This in turn may have prevented a four-day delay of clozapine titration. Earlier and more frequent CRP measurements titration would have also guided clinical assessment as well. Furthermore, this case stresses the larger implications of investigating medical comorbidities among patients presenting on a psychiatric unit.
